# Unified Modeling of Familial Mediterranean Fever and Cryopyrin Associated Periodic Syndromes

**DOI:** 10.1155/2015/893507

**Published:** 2015-05-28

**Authors:** Yasemin Bozkurt, Alper Demir, Burak Erman, Ahmet Gül

**Affiliations:** ^1^Computational and Quantitative Biology Lab, Koc University, 34450 Istanbul, Turkey; ^2^Division of Rheumatology, Department of Internal Medicine, Istanbul Faculty of Medicine, Istanbul University, 34093 Istanbul, Turkey

## Abstract

Familial mediterranean fever (FMF) and Cryopyrin associated periodic syndromes (CAPS) are two prototypical hereditary autoinflammatory diseases, characterized by recurrent episodes of fever and inflammation as a result of mutations in *MEFV* and *NLRP3* genes encoding Pyrin and Cryopyrin proteins, respectively. Pyrin and Cryopyrin play key roles in the multiprotein inflammasome complex assembly, which regulates activity of an enzyme, Caspase 1, and its target cytokine, IL-1*β*. Overproduction of IL-1*β* by Caspase 1 is the main cause of episodic fever and inflammatory findings in FMF and CAPS. We present a unifying dynamical model for FMF and CAPS in the form of coupled nonlinear ordinary differential equations. The model is composed of two subsystems, which capture the interactions and dynamics of the key molecular players and the insults on the immune system. One of the subsystems, which contains a coupled positive-negative feedback motif, captures the dynamics of inflammation formation and regulation. We perform a comprehensive bifurcation analysis of the model and show that it exhibits three modes, capturing the Healthy, FMF, and CAPS cases. The mutations in Pyrin and Cryopyrin are reflected in the values of three parameters in the model. We present extensive simulation results for the model that match clinical observations.

## 1. Introduction

Inflammation is the organisms' protective response to remove the insult and initiate the healing. Inflammatory reaction triggered by the recognition of pathogen or damage associated molecular patterns (PAMP or DAMP) usually results in the elimination of the stimuli and protection of the body integrity. Inflammasomes, multiprotein oligomers, form a link between the sensing of microbial products and developing an immune response to the danger signals via the regulation of the secretion of proinflammatory cytokines [1]. Inflammasome activation is highly regulated and the level of activation is critical to generate a proper immune response [2]. However, when the insult level is higher than the immune system can handle or due to problems in the control mechanisms regulating the reaction against these harmful stimuli, inflammatory disorders arise leading to various forms of discomfort, tissue or organ damage, or even death, depending on the magnitude of the insult or the nature of the insufficiency in the control mechanisms.

Quantitative approaches have recently been used in order to attain a better understanding of the immune system and immunity related diseases. Quantitative models have been used in order to understand how the immune cells and key molecular players interact with each other to constitute the total activity of the immune system [3]. Mathematical models are, of course, not yet detailed enough to describe the quantitative behavior of all immune cells and molecular species involved. However, the overall observed clinical behaviors can be modeled using several well established tools of chemical kinetics and dynamical systems theory. These models can then help explain observed clinical behavior and may further be used in designing custom drug therapies for immune system related diseases.

Autoinflammatory syndromes, also called hereditary periodic fever syndromes, are a class of disorders characterized by recurrent episodes of inflammation in tissues such as joints, skin, gut, and eyes, generally accompanied by fever, in the absence of any adaptive immune response by cytotoxic T cells or pathogenic autoantibodies [4]. Familial mediterranean fever (FMF) and Cryopyrin associated periodic syndromes (CAPS), which we consider in this paper, are among the prototypical members of this autoinflammatory disease class. FMF is caused by inherited loss-of-function mutations in Pyrin, and CAPS by gain-of-function mutations in Cryopyrin, two proteins that play key roles in the control and regulation of inflammation along with an enzyme, Caspase 1, and its target cytokine, IL-1*β*. Normally, triggered by a microbial or a sterile insult, Cryopyrin forms an inflammasome that converts precursor procaspase 1 into active Caspase 1. Once activated, Caspase 1 proteolytically cleaves proIL-1*β* into an active IL-1*β* with a proinflammatory effect [5]. Pyrin, on the other hand, acts as an anti-inflammatory mediator that controls and prevents Cryopyrin inflammasome formation by interacting with both Caspase 1 and Cryopyrin protein (Nlrp3). Mutated Pyrin can no longer act as an effective suppressor of inflammasome formation [6]. Mutations in Cryopyrin, on the other hand, result in elevated inflammasome formation even in the absence of a trigger or insult [7]. Overproduction of IL-1*β* by Caspase 1 as a result is the main cause of fever and inflammatory episodes in FMF and CAPS [8].

In this paper, we present a unifying dynamical model for FMF and CAPS in the form of coupled nonlinear ordinary differential equations. The model is composed of two subsystems. The first subsystem captures the interactions and dynamics of Pyrin, Cryopyrin, procaspase 1, Caspase 1, and inflammasome formation, including the effect of triggers. The second subsystem, which contains a coupled positive-negative feedback motif, captures the dynamics of IL-1*β*, its receptor, and receptor antagonist, including Caspase 1 independent cleavage of IL-1*β*. The two subsystems are coupled via the key player Caspase 1. We perform a comprehensive bifurcation analysis of the model and show that it exhibits three modes, capturing the Healthy, FMF, and CAPS cases. The mutations in Pyrin and Cryopyrin are reflected in the values of three parameters in the model. We then present extensive simulation results for the model, which match clinical observations. In the presence of a trigger, there is a normal increase in inflammation initiators in the Healthy mode. In FMF, the response to trigger introduction is more intense and a severe inflammatory cascade is activated. In CAPS, on the other hand, even in the absence of a trigger, periodically recurring, severe inflammatory episodes are observed. The proposed model also explains why a procaspase 1 inhibitor can be effective in treating FMF, but not CAPS, for which drugs that directly inhibit IL-1*β* are used.

The paper is organized as follows. First, background information on FMF and CAPS pathogeneses is given based on an extensive literature review. Next, the unified model that captures key aspects of FMF and CAPS is introduced. Detailed bifurcation analyses are performed on the model. Simulation results obtained from the proposed model are presented and discussed. Finally, conclusions are drawn.

## 2. Biological Background on FMF

FMF is a hereditary autoinflammatory disease associated with mutations in the* MEFV* (mediterranean fever) gene [9].* MEFV* gene is located in the short arm of chromosome 16 and encodes a 781-amino acid protein called Pyrin [10]. FMF is inherited in an autosomal recessive fashion (although there are some reported heterozygotes manifesting FMF) and mostly affects people originating from around the Mediterranean Sea, for example, mostly Armenians, Jews, Turks, and Arabs [11]. The main symptoms are irregular attacks of fever (38–41°C), abdominal pain, and chest and joint pain. The duration of the flare (1–3 days) and remission (weeks or months) period varies considerably. The acute phase response includes an increase in the white blood cell count, erythrocyte sedimentation rate, fibrinogen, C-reactive protein, serum amyloid A, and phospholipase A_2_ [12]. Acute phase response increases during the attack and decreases in the attack-free period.

Although Pyrin's structure and function have not been completely elucidated yet, it is clear that it has a role in the control of the production of IL-1*β* and other proinflammatory cytokines. Mutations in exon 10 of the* MEFV* gene leads to ineffective Pyrin function. Pyrin is expressed mainly in neutrophils, eosinophils, monocytes, dendritic cells, and synovial and peritoneal fibroblasts (mostly in the innate immune system cells) and regulates Caspase 1 activation [13]. Caspase 1 is responsible for cleavage of proIL-1*β* and the subsequent release of bioactive IL-1*β* [5]. IL-1*β* is a very important pyrogenic cytokine in the inflammatory process. In FMF, overstimulation of IL-1*β* production causes increased inflammatory and febrile response.

**Figure 1:**
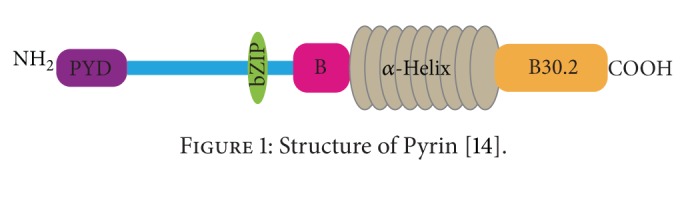
Structure of Pyrin [14].

Pyrin is composed of 5 domains: Pyrin (PYD), bZIP transcription factor basic, B-box zinc finger, *α*-helical (coiled coil), and B30.2 (PRY-SPRY) domains [14]. The structure of Pyrin is shown in Figure 1.

Cryopyrin inflammasome, inflammasome of interest in FMF, is a multiprotein oligomer containing ASC, procaspase 1, Cryopyrin, and Cardinal proteins [15]. Inflammasome formation takes place, depending on the type of the inflammasome, in response to the specific pathogen associated molecular patterns (PAMP) such as lipopolysaccharide (LPS), dsRNA, and peptidoglycan or damage associated molecular patterns (DAMP) such as ATP and uric acid. This protein complex regulates and proteolytically activates important cytokines such as IL-1*β* and IL-18 through the activation of Caspase 1 [16].

Cryopyrin protein is encoded by the* NLRP3* gene and belongs to the nucleotide-binding oligomerization domain receptors (NLR) protein family. Cryopyrin can detect a variety of danger signals such as dsRNA, uric acid, bacterial ligands, and imidazoquinolines [17]. PYD is a common domain found in proteins such as Pyrin, Cryopyrin, and some other NLR proteins. This domain allows the homotypic interaction with other proteins containing PYD. For example, since ASC contains a PYD, Pyrin and Cryopyrin interact with ASC through this domain. Cryopyrin is an essential component of the Cryopyrin inflammasome.

**Figure 2:**
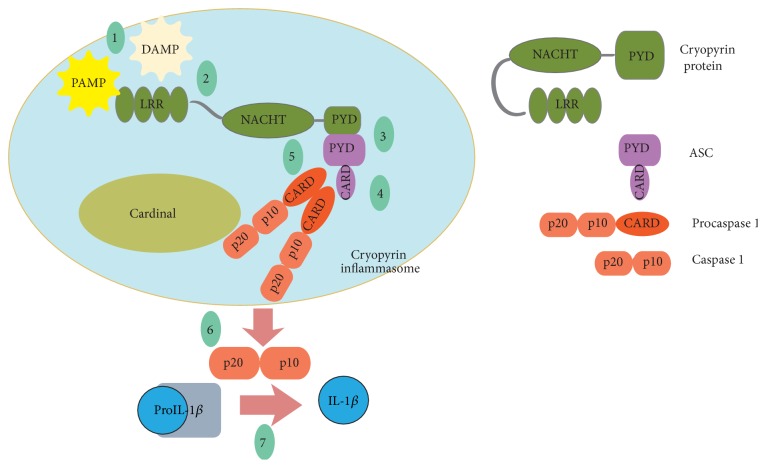
Cryopyrin inflammasome formation steps. (1) With DAMP or PAMP, inflammasome formation process is triggered. (2) A conformational change occurs in Cryopyrin protein due to the trigger. (3) Interaction between PYDs of ASC and Cryopyrin protein becomes possible after the conformational change. (4) CARD of ASC and procaspase 1 interact. (5) Cardinal brings another procaspase 1 to the system. (6) Induced proximity mediated autocatalysis results in the activation of Caspase 1. (7) Caspase 1 bioactivates IL-1*β* by cleavage.

Without any trigger (DAMP or PAMP), interaction between PYD of Cryopyrin and ASC is not possible due to the nominal structure of Cryopyrin. With the presence of a stimulus, the interaction of the PYD domain of Cryopyrin and ASC becomes possible. ASC interacts with the PYD of Cryopyrin through its N terminal PYD. C terminal CARD domain of ASC is in interaction with the CARD domain of procaspase 1. The Cardinal brings another procaspase 1 to the system. Procaspase 1 molecules become closer to each other (proximity-induced-mediated-autocatalysis) which results in the proteolytic activation and release of active Caspase 1 protein with two chains of p20 and p10 [14]. ProIL-1*β* is a precursor which is proteolytically cleaved to a shorter, active form, IL-1*β*, by Caspase 1.  Figure 2 summarizes the inflammasome formation steps. Mature IL-1*β* molecules induce expression of other cytokines. Secondary cytokines such as IL-6 recruit immune cells to the site of inflammation. There is a balance between the levels of activation and inhibition of inflammasome formation. Although IL-1*β* molecules help combat the infection, spontaneous or triggered overproduction of IL-1*β* causes adverse effects that are associated with inflammatory diseases such as FMF and CAPS.

There is evidence for both the anti-inflammatory [6, 14, 18] and proinflammatory roles of Pyrin [19, 20]. Anti-inflammatory role includes the inhibition of Caspase 1 action and IL-1*β* processing. Proinflammatory role portrays Pyrin as a constituent of an unknown inflammasome resulting in more IL-1*β* activation. Since there is more literature for the suppressive effect of Pyrin on inflammation, throughout this paper, we will take Pyrin as an anti-inflammatory mediator.

Some of the mechanisms on the contribution of Pyrin in inhibiting Caspase 1 activation are listed here.

**Figure 3:**
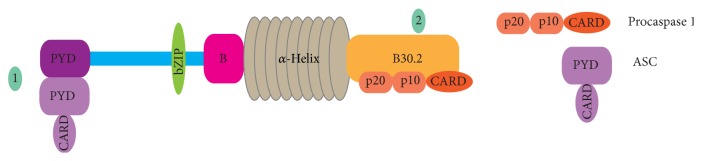
Anti-inflammatory role of Pyrin.

- ASC binds to Pyrin through its PYD. Thus, ASC cannot participate in the formation of the Cryopyrin inflammasome. Since Cryopyrin inflammasome assembly does not take place, Caspase 1 is not activated, and hence IL-1*β* is not produced (step 1 in Figure 3).
- Due to the interactions between B30.2 domain of WT Pyrin and the p20 and p10 domains of Caspase 1, Pyrin can directly bind to both procaspase 1 and Caspase 1 and hence indirectly prevent IL-1*β* activation. In FMF, the most common mutations (M608I, M694V, and V726A) are generally in the C terminal B30.2 domain of Pyrin. If there is a mutation in B30.2 domain of Pyrin, the interaction between this domain and p20 and p10 subunits of Caspase 1 is diminished [6]. p20 and p10 form a heterodimer; that is, Caspase 1 becomes activated (step 2 in Figure 3).
- Pyrin also interacts directly with proIL-1*β* which is an additional inhibition factor on IL-1*β* secretion.

Maturation and secretion of IL-1*β* require two distinct signals, priming and activation [21]. With the priming signal, transcription and translation of proIL-1*β* take place. Activation signal results in the assembly of Cryopyrin inflammasome, Caspase 1 activation, and subsequently IL-1*β* activation and release.

**Figure 4:**
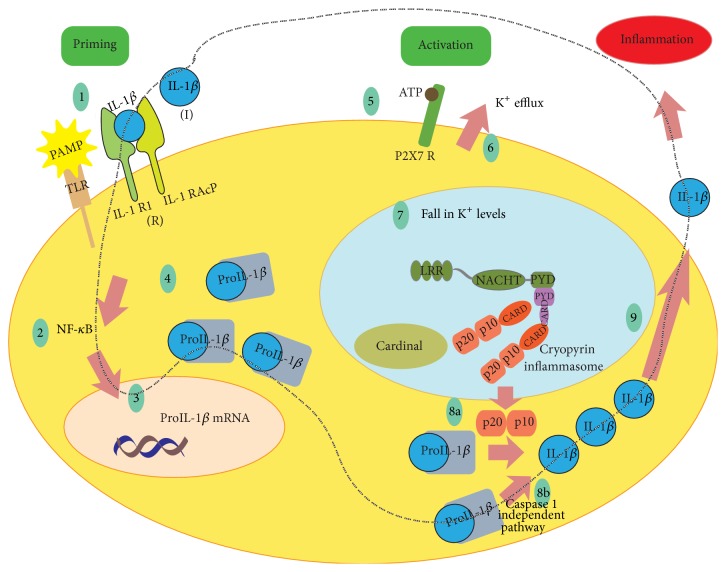
Maturation and secretion of IL-1*β* requires two signals. Binding of ligand (PAMP or IL-1*β*) to TLR (1) activates NF-*κ*B pathway (2). NF-*κ*B signaling results in proIL-1*β* transcription and secretion (3 and 4). Binding of ATP to the P2X7 receptor is considered as the activation signal (5) which causes K^+^ efflux (6) and subsequently a decrease in intracellular K^+^ concentration (7). Together with PAMP or DAMP, fall in K^+^ acts as the initiator of the inflammasome formation (7). ProIL-1*β* is cleaved by the product of the inflammasome, Caspase 1 (8a). However, Caspase 1 is not the only protease that can cleave proIL-1*β* (8b). Mature IL-1*β* is secreted and inflammation process is then started (9). Dashed path shows the positive feedback relationship between the free IL-1*β* (I) and bound IL-1*β* (R).

IL-1R1 is a member of TLR superfamily [22]. Binding of mature IL-1*β* to IL-1R1 results in the downstream signaling of NF-*κ*B pathway leading to the production of proIL-1*β* [23]. Also, recognition of the microbial ligands by TLRs initiates proIL-1*β* transcription. Microbial ligands and endogenous cytokines are the priming signals for proIL-1*β* production (step 1 in Figure 4).

Activation signal (such as ion/membrane perturbations, reactive oxygen species (ROS), pore-forming toxins, crystals, and ATP) promotes the indirect activation of IL-1*β* secretion [24]. In Figure 4, ATP activating Cryopyrin inflammasome is shown as a representative example. Activation of P2X7 receptor by extracellular ATP results in the K^+^ efflux. Fall in the intracellular K^+^ levels triggers Cryopyrin inflammasome formation [25]. Inflammasome oligomerization leads to activation of Caspase 1, followed by the maturation and secretion of IL-1*β*. We should also note here the possible effect of other proteases. Caspase 1 specifically cleaves 31-kd precursor proIL-1*β* to 17-kd biologically active IL-1*β*. Caspase 1 belongs to the caspase family of cysteine proteases. Some of the other members of protease family, such as neutrophil serine proteases, proteinase 3, and mast cell derived serine proteases, can also cleave proIL-1*β* [26]. This suggests a redundancy in the mechanisms of IL-1*β* processing. The role of other proteases is much more apparent in some models of inflammatory diseases such as arthritis animal models [27]. Even though in FMF and CAPS overproduction of IL-1*β* is mostly related to the high levels of Caspase 1 concentrations, we will also include the possible effect of other proteases in the model.

In Figure 4, the dashed path shows the positive feedback relationship between the bound IL-1*β* (R) and free IL-1*β* (I). Binding of IL-1*β* to its receptor IL-1R1 results in more proIL-1*β* transcription and thus more IL-1*β*. In FMF patients, inflammasome activation is higher when compared to the healthy patients [6]. Overactivation of inflammasome causes overproduction of IL-1*β*. This process is partially self-sustaining; that is, IL-1*β* causes more IL-1*β* production (either via Caspase 1 or through Caspase 1 independent pathway) since IL-1*β* is one of the primary signals for IL-1*β* maturation and secretion.

IL-1*β* and other proinflammatory innate cytokines are the main mediators of autoinflammatory diseases. The mechanism for the cytokine induction pathway is not fully clear. FMF symptoms develop as a result of mutations disrupting functions of Pyrin, which result in overproduction of IL-1*β*. However, not only IL-1*β* but also a downstream cytokine IL-6 is essential in attack and fever development in FMF. High levels of IL-1*β* cause an increase in IL-6 [28].

IL-6 has both proinflammatory and anti-inflammatory roles. IL-6 changes the fever set point in the hypothalamus, responsible for the high fever in the attack period of FMF. Additionally, it mediates acute phase proteins. Levels of leukocyte count, ESR, CRP, sIL-2R, IL-6, and IL-10 increase considerably during an attack [29]. High levels of IL-6 may have suppressive effect in the production of other cytokines such as IL-1*β* and TNF-*α* while activating the antagonist of IL-1*β*, IL-1Ra [30].

IL-1*β* is the most critical endogenous pyrogen in autoinflammatory diseases and its control is extremely important. Its receptor binding gives some clues regarding the possible mechanisms that may lead to ending the active phase in FMF. There are natural inhibitors of IL-1 such as IL-1Ra, decoy receptor of IL-1R2, and other soluble receptors. In knock-out mice lacking IL-1Ra, excessive inflammation has been observed. These mice develop spontaneous joint inflammation, vasculitis, and skin inflammation [31]. The biological activities of IL-1 are initiated by binding of IL-1*α* and/or IL-1*β* to the same receptor, namely, IL-1R1. IL-1R1 exists on the surface of a wide variety of cells. Binding of IL-1*α* and/or IL-1*β* causes a conformational change in IL-1R1 and recruits an accessory protein, IL-1RAcP [32]. Once IL-1/IL-1R1/IL-1RAcP complex is formed, signaling through other cascades such as NF-*κ*B is activated. This is the only active form of this complex. Other scenarios fail to generate an active signal. Some of the cases that may cause no signal are listed here.

- *IL-1Ra.* It competes with IL-1*β* for binding to its receptor IL-1R1 and prevents binding of IL-1*β* and the subsequent downstream signaling [33].
- *IL-1R2.* It is a decoy receptor similar to IL-1R1. IL-1*β* binds to IL-1R2 instead of IL-1R1. Cascades of other cytokines are not activated when IL-1*β* does not bind to IL-1R1 and transmit downstream signals [34].
- *SIGIRR.* It prevents IL-1R1/IL-1RAcP heterodimerization [35].
- *Soluble IL-1R1 or R2*. They are soluble receptors that can bind to IL-1 and IL-1RAcP but are incapable of propagating a signal [36].

## 3. Biological Background on CAPS

CAPS comprises a spectrum of rare autoinflammatory syndromes, ranging from FCAS (familial cold autoinflammatory syndrome) and MWS (Muckle-Wells syndrome) to NOMID (also called as CINCA) (neonatal-onset multisystem inflammatory disease). These diseases are caused by autosomal dominantly inherited gain-of-function or* de novo* mutations in various domains of* NLRP3* gene (also known as* CIAS1* gene), located on chromosome 1 (1q44), which encodes a Pyrin like protein, Cryopyrin (or Nlrp3 protein). Approximately 100 different mutations (mostly missense mutations) in exon 3 of this gene have been identified. Cryopyrin is expressed in monocytes, neutrophils, and chondrocytes [37]. Cryopyrin is one of the NLR family proteins with a critical role in the regulation of the inflammatory response. CAPS, or Cryopyrinopathies, lead to increased and spontaneous activity of Nlrp3-associated Caspase 1 activating inflammasome, that is, Cryopyrin inflammasome. The more inflammasome formation takes place, the more conversion of proIL-1*β* into IL-1*β* occurs. IL-1*β* not only activates the fever pathway, but also causes pain sensitization and bone and cartilage destruction and activates acute phase response [38]. Although the initial steps of the pathogenesis of FMF and CAPS are different, they become quite similar in terms of the main causative pathway and some of the design strategies in the treatment. IL-1 blocking therapies are applied to CAPS patients as well as colchicine-resistant FMF patients successfully. Similarities between FMF and CAPS pathogeneses are not repeated in this section; only the differences are indicated instead.

In Table 1, CAPS types and their symptoms are listed. The severity of the disease is also indicated.

Although CAPS attacks might occur following the initiation of a stimulus such as cold exposure in the mildest form of CAPS spectrum, that is, FCAS, no trigger is identified or associated in most disease attacks in more severe forms of MWS and NOMID [39]. This suggests that CAPS arise as recurrent episodes of fever even in the absence of any insult due to spontaneous activation of Cryopyrin inflammasome, and, in severe cases such as NOMID, an episode-free continuous inflammation can be observed. How exposure to cold in patients with FCAS induces the inflammatory disease flares remains unknown [39].

Similar symptoms and characteristics with different severity among FCAS, MWS, and NOMID suggest that it is possible to capture all of the spectrum in the same model.

## 4. FMF and CAPS Pathogeneses with a Modeling Perspective

The external causes triggering FMF attacks were studied and tested thoroughly [16, 40]. CAPS flares, on the other hand, seem to be self-sustaining. At least, triggers for CAPS have not been identified in the same extent as for FMF. This suggests that FMF attacks occur as a result of yet unknown external or endogenous triggers. The triggers (PAMP or DAMP) activate inflammasome formation and subsequently overproduction of IL-1*β* due to malfunctioning Pyrin in FMF patients, as described in Section 2. The immune response is then activated and the disease symptoms are observed in the attack period. When the trigger is deactivated by the immune action, disease enters a quiescent phase, where most of the disease symptoms disappear. CAPS attacks, on the other hand, seem to be related to the autonomous dynamic properties of the key players' relative concentrations. Positive and negative feedback mechanisms cannot keep these concentrations in normal ranges as a result of the mutations in Cryopyrin, which causes periodic flare/remission cycles.

In FMF and CAPS, mutations in the wildtype proteins (Pyrin and Cryopyrin) result in overproduction of IL-1*β*, stimulating an inflammatory response. As pointed out in Sections 2 and 3, interactions between positive and negative feedback mechanisms determine the overall behavior. Coupled positive and negative feedback loops are a widely seen motif that can exhibit various types of dynamics [41]. Here, we summarize important positive and negative feedback interactions that take part in both FMF and CAPS.

*Positive Feedback Mechanisms*

- Signaling by Receptor-IL-1*β* complex increases IL-1*β* levels, through further transcription of proIL-1*β*.
- Additionally, Caspase 1 independent processing of IL-1*β* increases the active Receptor-IL-1*β* complex level.

*Negative Feedback Mechanisms*

- Receptor-IL-1*β* complex also induces the expression of the antagonist.
- Binding of the antagonist to the receptor does not result in IL-1*β* signaling and hence decreases the active Receptor-IL-1*β* complex levels.

The key variables in FMF and CAPS pathogeneses are as follows.

- Trigger (T): it represents PAMP and DAMP which activates Cryopyrin inflammasome formation.
- IL-1*β* (I): it indicates the serum levels of IL-1*β*.
- Antagonist (A): it represents the total activity that decreases the binding of IL-1*β* to its receptor (e.g., IL-1Ra, sIL-1R2).
- Receptor (R): it is used to indicate the amount of bound IL-1*β*, that is, receptor-IL-1*β* complex.
- Caspase 1 (C): it is Caspase 1 level.
- Procaspase 1 (PC): it represents the free (not-Pyrin bound) procaspase 1 levels.
- Pyrin (P): it represents the amount of Pyrin (assumed to be constant).

**Figure 5:**
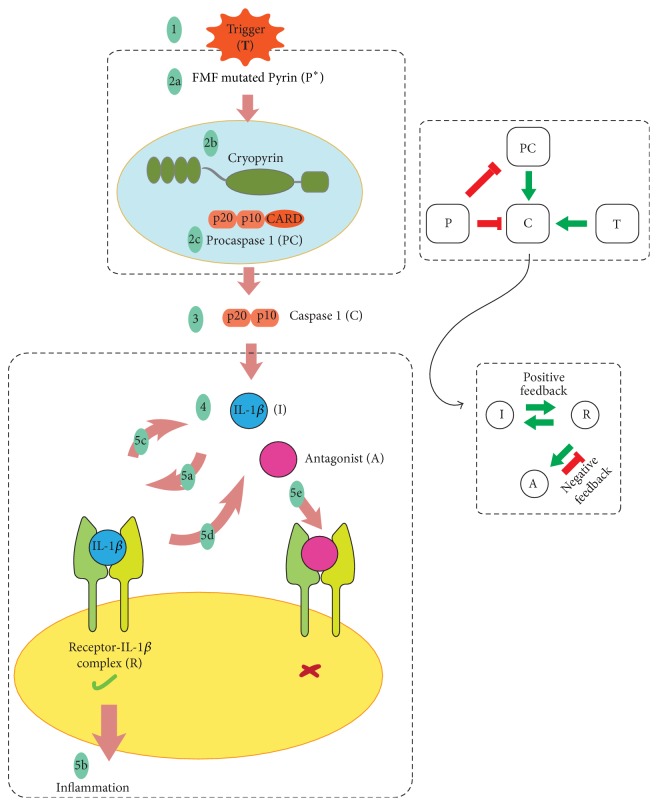
Summary of the pathogenesis of FMF. With trigger, inflammasome formation process is triggered in Pyrin mutants (1 and 2a). Cryopyrin protein and procaspase 1 complex into an inflammasome due to nonfunctioning Pyrin (2b and 2c). Procaspase 1 turns into Caspase 1 by the inflammasome action (3). IL-1*β* is maturated by Caspase 1 (4). IL-1*β* binds to the receptor (5a). IL-1*β* signaling cascade followed by inflammation is activated by the binding of IL-1*β* (5b). Signaling by the Receptor-IL-1*β* complex leads to further transcription of proIL-1*β*, resulting in an increase in IL-1*β* levels. Here, we had only considered the Caspase 1 independent processing of IL-1*β* (5c). Signaling by the Receptor-IL-1*β* complex also stimulates the antagonist production (5d). Binding of antagonist to the receptor does not result in active IL-1*β* signaling (5e).

**Figure 6:**
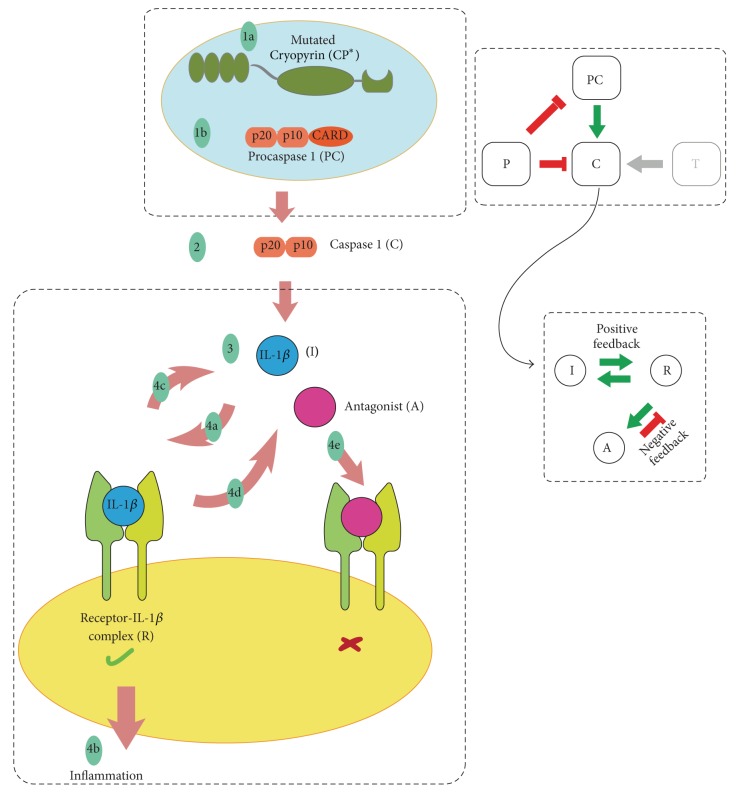
Summary of the pathogenesis of CAPS. Even without the trigger, inflammasome formation process takes place in Cryopyrin mutants (1a and 1b). Procaspase 1 turns into Caspase 1 by the inflammasome action (2). IL-1*β* is maturated by Caspase 1 (3). IL-1*β* binds to the receptor (4a). IL-1*β* signaling cascade followed by inflammation is activated by the binding of IL-1*β* (4b). Signaling by the Receptor-IL-1*β* complex leads to further transcription of proIL-1*β* and thus increase in Caspase 1 independent IL-1*β* levels (4c). Signaling by the Receptor-IL-1*β* complex also stimulates the antagonist production (4d). Binding of antagonist to the receptor does not result in active IL-1*β* signaling (4e).

We consider overproduction of IL-1*β* as the driving cause of inflammation. PAMP and DAMP are taken as triggers which activate inflammasome formation and the subsequent processes in Pyrin mutants. When trigger is not present, FMF is in its quiescent phase. The action of the regulators of IL-1*β* levels (IL-1Ra, sIL-1R2, etc.) is lumped and considered as one variable, the antagonist A. Figures 5 and 6 illustrate the most crucial steps we have identified in FMF and CAPS pathogeneses which form the foundation of the mathematical model that will be described in Section 5.

## 5. Modeling of FMF and CAPS

Motivated by the considerations in Sections 2, 3, and 4, we construct a unifying disease model which captures both FMF and CAPS. Parameters used in the model are listed in Table 2.

The kinetic equations are given in (1a), (1b), (1c), (1d), and (1e) in the form of ordinary differential equations (ODEs) that describe the interactions of the key variables involved. The kinetic system is formed as a composition of competitive and noncompetitive inhibition models used in biochemistry [42]. We follow a similar approach in model development as in [41] for the coupled positive and negative feedback processes. Here, to represent the interactions between R, I, and A, we utilize competitive inhibition, due to the fact that the antagonist also binds to the same receptor and is structurally similar to the substrate, IL-1*β*. The inflammasome formation process is modeled based on noncompetitive inhibition reactions. Procaspase 1 is considered as a substrate. Pyrin, on the other hand, inhibits inflammasome formation by binding to PC and C. The product of the process is C. Hill effect is also taken into consideration in both inhibition reactions [43]. Consider

(1a) $$\begin{array}{c} \frac{d\left[R\right]}{dt}=V_{r}\cdot \frac{{\left(\left[I\right]/K_{ir}\right)}^{n}}{1+{\left(\left[I\right]/K_{ir}\right)}^{n}+{\left(\left[A\right]/K_{ar}\right)}^{n}}-k_{dr}\cdot \left[R\right]+k_{br}, \end{array}$$

(1b) $$\begin{array}{c} \frac{d\left[I\right]}{dt}=V_{i}\cdot \frac{{\left(\left[R\right]/K_{ri}\right)}^{n}}{1+{\left(\left[R\right]/K_{ri}\right)}^{n}}-k_{di}\cdot \left[I\right]+\alpha \cdot \left[C\right], \end{array}$$

(1c) $$\begin{array}{c} \frac{d\left[A\right]}{dt}=V_{a}\cdot \frac{{\left(\left[R\right]/K_{ra}\right)}^{n}}{1+{\left(\left[R\right]/K_{ra}\right)}^{n}}-k_{da}\cdot \left[A\right]+k_{ba}, \end{array}$$

(1d) dCdt=Vc·PC/Kpccn1+PC/Kpccn·T/Ktcn1+T/KtcnW·11+P/Kpcn−kdc·C+kbc,

(1e) dPCdt=Vpc·11+P/Kppcn−kcW·C−kdpc·PC+kbpc.

For all the variables (R, I, A, C, and PC) we have assumed a basal synthesis rate. Since the production rate of I is directly related to the C concentration according to our model, basal synthesis rate of I is not included explicitly; that is, *k*_*bi*_ does not appear in (1b). The rate of degradation for a certain variable is assumed to be proportional to the concentration of that variable.

Equation (1a) captures competitive inhibition reactions where I is the substrate and A is the inhibitor. Equation (1b) represents the effect of Caspase 1 independent positive feedback mechanism between R and I. Activation of A by R is captured in (1c).

C, on the other hand, is generated as a result of a noncompetitive binding reaction as represented by (1d). Here, binding is not used in a strict sense, instead it captures the possible interactions. With the initiation of T, PC forms the inflammasome at a maximal rate of *V*_*c*_. P inhibits inflammasome formation since it binds to PC and C. In inflammasome formation, other proteins such as Cryopyrin, ASC, and Cardinal concentrations are taken as constant and not represented explicitly. Conversion of PC into C indicates the formation of the inflammasome. Decrease in PC levels due to conversion to C is captured in (1e) with a rate constant *k*_*c*_. C sets the basal synthesis rate for I proportional to a constant *α*.

### 5.1. Selection of Parameter Values and Capturing Mutations

The proposed model captures Healthy, FMF, and CAPS cases in a unified manner, via changes in the values of only three model parameters, without the addition or removal of equations to/from the model. In Healthy mode, nominal values of the parameters that are listed in Table 3 are used. Some of these nominal parameter values were simply normalized to 1; others were chosen based on the model parameter values given in [41] and by running extensive bifurcation analyses (described in Section 6) and parameter sweeps. The parameter values were chosen in such a way so that the characteristic and clinical features of both diseased FMF and CAPS systems, along with the healthy system, can be observed in the same model, by modifying only a small subset of the parameters. FMF is a result of a mutation in Pyrin. This mutation is attributed to an increase in the threshold for P to suppress C and PC, captured by two parameters *K*_*pc*_ and *K*_*ppc*_ in the model. The values of these two parameters are increased to 10 from their nominal value at 1 in order to reflect the FMF mutation. CAPS is associated with a mutation in Cryopyrin and subsequently increased inflammasome formation. This mutation is captured by the parameter *V*_*c*_, which controls the rate of inflammasome formation in the model. The value of this parameter was increased to 450 from its nominal value of 1.15 in order to reflect the CAPS mutation and observe the clinical features of CAPS in the model. Various members of the CAPS disease family (FCAS, MWS, and NOMID) can also be captured by the model via changes in the value of *V*_*c*_ and by setting the trigger level T to an appropriate level.

### 5.2. The Model as a Composition of Two Subsystems

**Figure 7:**
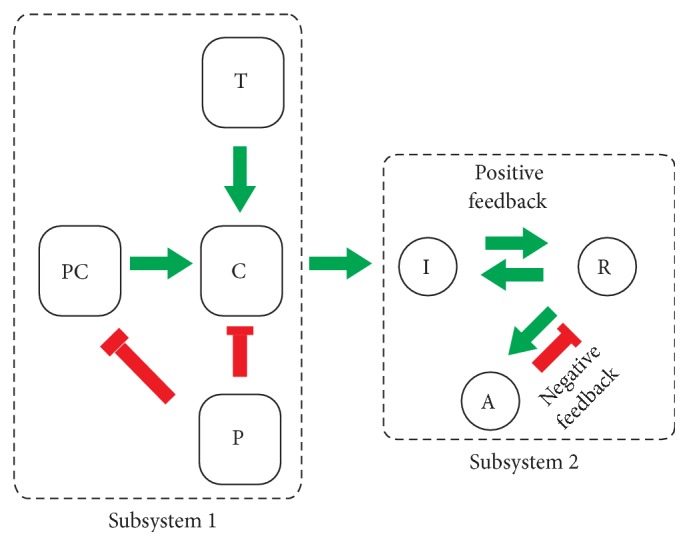
The model as a composition of two subsystems. The direction of each green (red) arrow represents a stimulation (inhibition) effect.

The model can be separated into two distinct subsystems as shown in Figure 7.* Subsystem 1* is composed of noncompetitive inhibition reactions among PC, T, P, and C and essentially determines the amount of Caspase 1. These interactions are captured by (1d) and (1e). Proportional to a constant *α*, C (Caspase 1) concentration then contributes to production of I. The only link between the competitive binding reactions among R, I, and A that make up* Subsystem 2* and* Subsystem 1* is via the C effect in I.* Subsystem 2* is represented by (1a), (1b), and (1c). Emerging as an input to* Subsystem 2* and produced by* Subsystem 1*, this model composed of two subsystems suggests that Caspase 1 level is the most critical parameter of the total system. Depending on the amount of Caspase 1 level,* Subsystem 2* exhibits varying characteristics, that is, monostability, excitability, and oscillatory behavior, which we analyze next in detail. These behaviors correspond to three distinct modes of the model, Healthy, FMF, and CAPS, respectively.

## 6. Bifurcation Analyses

The three distinct behaviors emerging from the model, Healthy, FMF, and CAPS, are revealed here with bifurcation analyses. Since Caspase 1 level is considered as the most critical component of the total system, it is selected as the bifurcation parameter. Caspase 1, being the output of* Subsystem 1*, has a constant, steady-state value that depends on the values of the model parameters; that is, it can be shown that* Subsystem 1* always has one stable fixed-point. This observation enables uncoupled analysis of* Subsytem 1* and* Subsystem 2*. We perform bifurcation analyses on* Subsystem 2* by considering the Caspase 1 concentration C as a bifurcation parameter that is swept in a certain range. One can think of each C value in this sweep to correspond to a stable fixed-point of* Subsytem 1* for a certain assignment to the model parameters of* Subsytem 1* and a certain trigger level as represented by T. The mutations in Pyrin and Cryopyrin are modeled as changes in the values of certain parameters in* Subsytem 1* as described before and result in an increase in the C as the output of* Subsytem 1*. In addition, higher values of trigger T also result in higher values for C.

**Figure 8:**
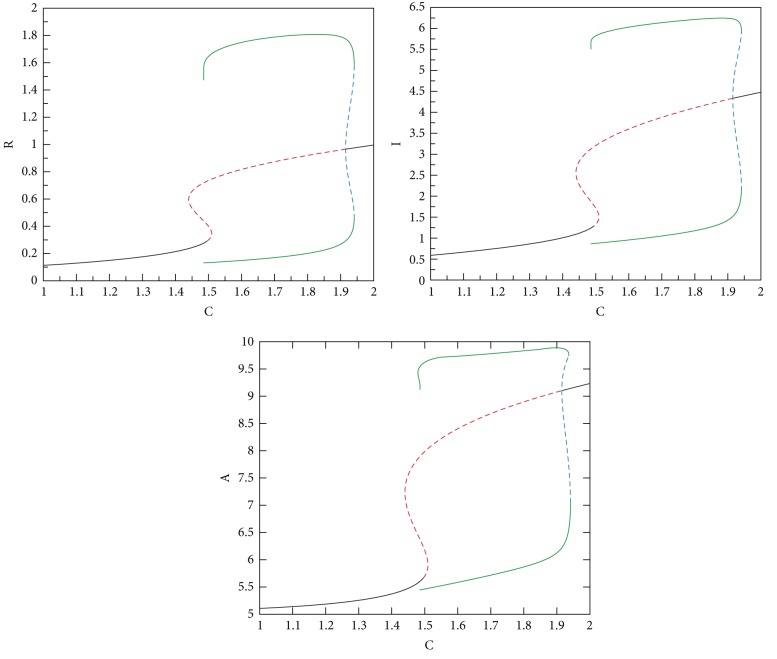
Bifurcation analyses. Stable (unstable) solutions are shown as a solid black (red dashed) line. Periodic stable (unstable) solutions are represented by green solid (blue dashed) lines.

In the bifurcation analysis we perform, as C is swept in a certain range, we determine the characteristics of the steady-state solutions of* Subsystem 2*. The results of this analysis are presented in Figure 8, where stable steady-state solutions are shown as a solid line and the unstable ones are represented by dashed lines. Periodic stable (unstable) solutions are shown by green solid (blue dashed) lines. The bifurcation diagrams for all* Subsystem 2* variables, that is, for R, I, and A, follow the same stability pattern.

**Figure 9:**
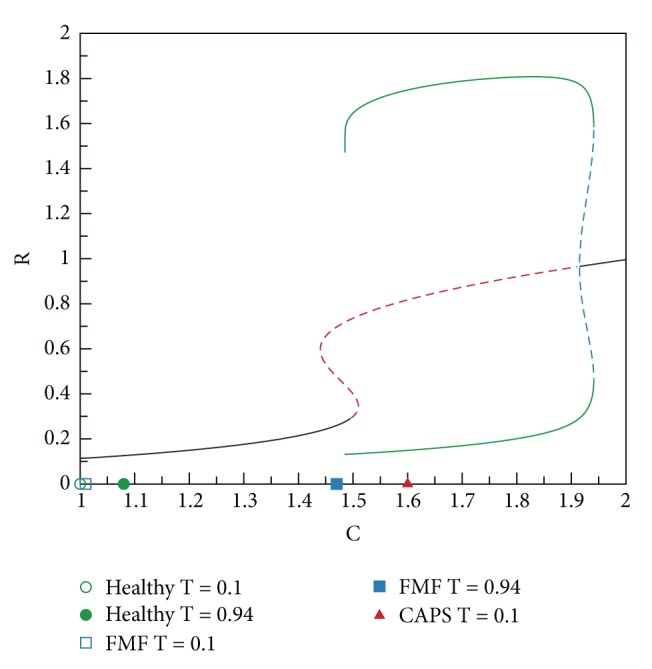
Classification of the three modes according to the bifurcation analysis.

In the three modes, the increase in C levels due to a pulse of trigger T with amplitude varying from 0.1 to 0.94 is shown in Figure 9. Nonzero low level of trigger T reflects the base trigger level which is always present in the environment. Classification of the three modes is as follows.

**Figure 10:**
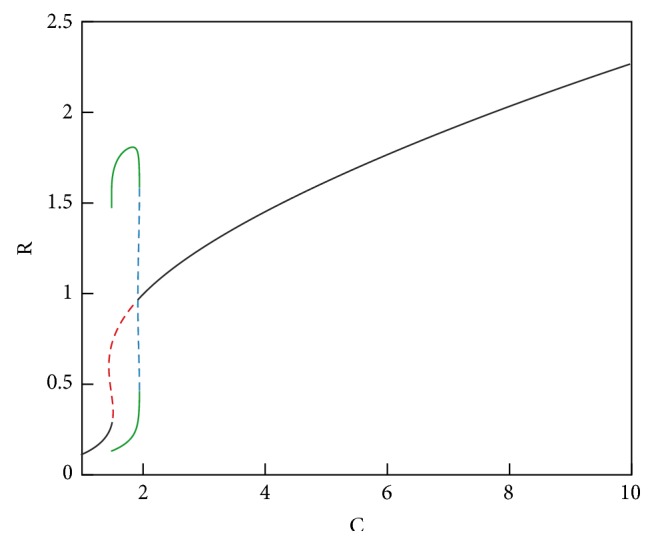
Extended bifurcation analysis.

- *Healthy (Monostable-Low) Mode.* For low values of C (C = 1 to C = 1.44),* Subsystem 2* has one stable fixed-point. A trigger pulse causes a slight increase in the C level in this range but does not result in a qualitative change in the characteristics of the solution of* Subsystem 2*, as seen in Figure 9. Increasing values of C in this region does not cause drastic changes in R, I, and A.
- *FMF (Excitable).* In the region between C = 1.44 and C = 1.48,* Subsystem 2* has two unstable fixed-points in addition to a stable fixed-point. When a trigger pulse pushes the system into this region, immune response becomes stronger compared with the ones observed for lower values of C. This becomes possible due to the effect of the Pyrin mutation which elevates the C level.
- *CAPS (Oscillatory and Monostable-High).* When C is increased further (C = 1.48 to C = 1.9) due to the type of Cryopyrin mutations,* Subsystem 2* has one stable periodic solution, that is, a limit cycle, and an unstable fixed-point. In this mode, limit cycle oscillations in R, I, and A are observed. In CAPS, C levels are always high due to the autosomal dominantly inherited mutations in Cryopyrin, resulting in oscillatory behavior even without a trigger pulse. The maximum levels observed for R, I, and A are the highest in this mode. Going beyond C = 1.9 corresponds to a more severe member of the CAPS disease family (NOMID), which is shown in Figure 10. In this range, oscillations (flare-remission cycles) disappear; there is one stable fixed-point. However, the values of R, I, and A stay at elevated levels indicating a much stronger immune response and hence continuous inflammation and fever.

Numerical bifurcation analyses presented in Figure 8 were performed with the XPPAUT software package [44]. Stability of the steady-state solutions was further verified by time-domain simulations using MATLAB [45].

## 7. Results and Discussion

**Figure 11:**
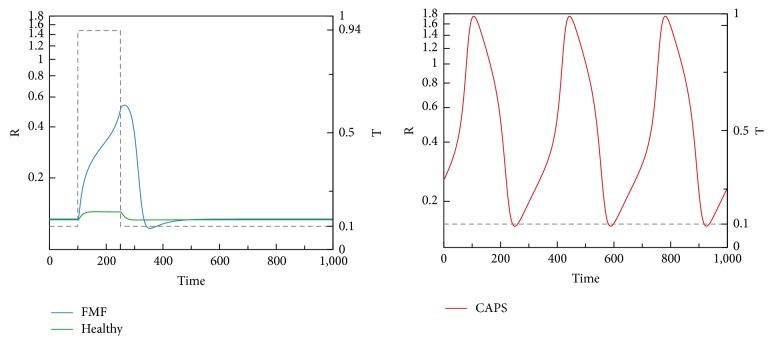
R levels in Healthy, FMF, and CAPS cases.

**Figure 12:**
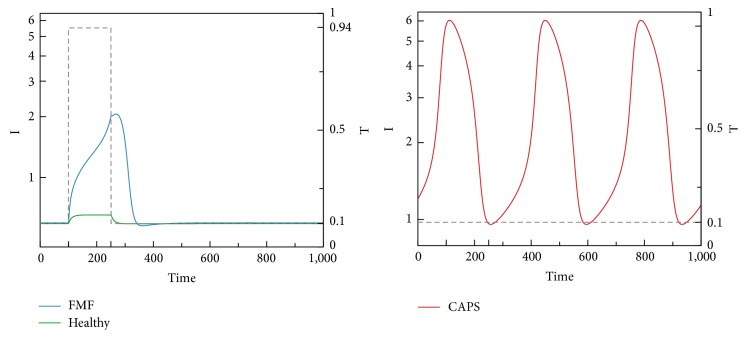
I levels in Healthy, FMF, and CAPS cases.

**Figure 13:**
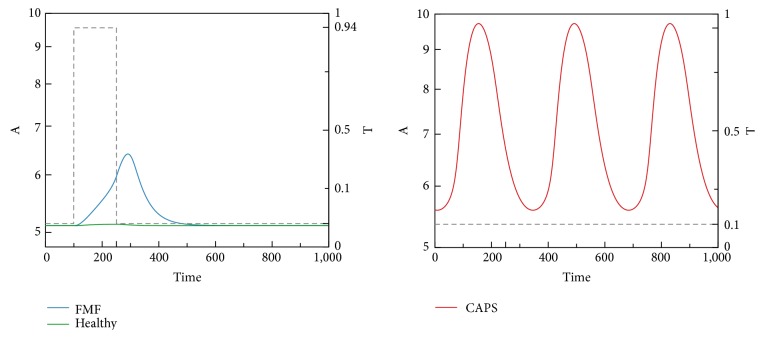
A levels in Healthy, FMF, and CAPS cases.

**Figure 14:**
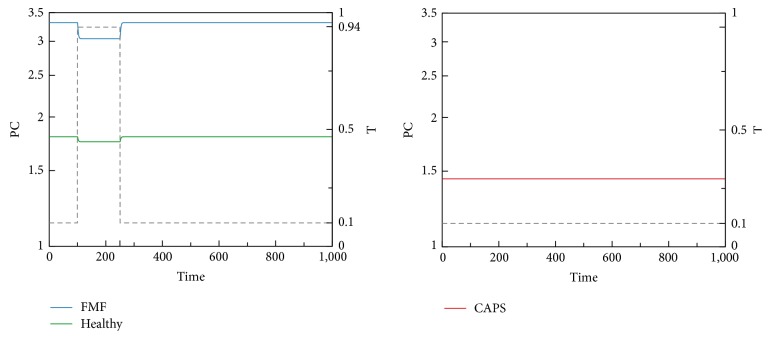
PC levels in Healthy, FMF, and CAPS cases.

**Figure 15:**
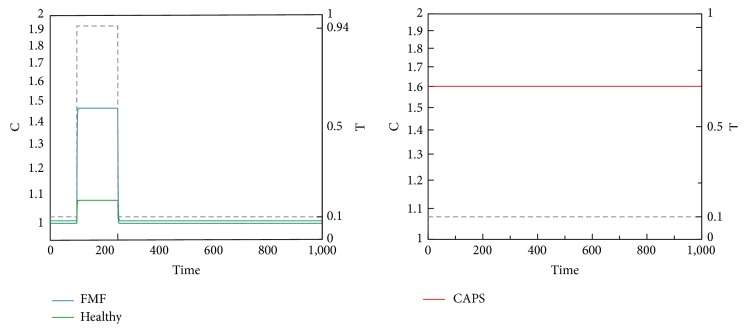
C levels in Healthy, FMF, and CAPS cases.

We now present time-domain simulations for R, I, A, PC, and C in response to a pulse trigger input in Healthy and FMF modes and a constant trigger in CAPS, as shown in Figures 11, 12, 13, 14, and 15. In Healthy and FMF modes, magnitude of T is stepped from 0.1 to 0.94 and back to 0.1. The trigger is applied at time = 100 and duration of the trigger pulse is set to 150. In CAPS, a constant base trigger level of T = 0.1 is applied. The dashed lines represent T in the plots.

Due to a pulse of trigger, there is a slight increase in R, in the Healthy mode. The increase in R is not large enough to activate the cytokine cascade for inflammation. In FMF, larger R values are observed, initiating the inflammation. When the trigger disappears, R values return to normal ranges in a while. High R values correspond to the flare (attack) period in FMF. When the trigger level is subdued, remission period continues. Depending on the magnitude and duration of the trigger pulse, attacks occur or do not occur, which fit well with the clinical experiences reflecting irregular periodicity of attacks ranging from weekly to ones that occur once in several months. In CAPS, even though T is kept at a low nominal value (T = 0.1), R levels oscillate due to the dynamics of the interaction between R, I, and A. The maximum R level is higher than that of FMF. As captured by our model, CAPS patients follow this oscillatory flare/remission pattern. I and A exhibit the same behavior as R.

Due to the initiation of triggers, procaspase 1 is converted to Caspase 1 through Cryopyrin inflammasome formation, as captured by* Subsystem 1*. Due to the mutation in Pyrin in FMF, Pyrin cannot suppress inflammasome formation by binding to PC. As shown in Figure 14, in FMF, PC level is the highest due to the mutation in Pyrin. In CAPS, as a result of the Cryopyrin mutation, conversion of PC to C is more favored. Therefore, PC is the lowest in the CAPS mode. Although FMF and CAPS are similar diseases in terms of inflammasome-associated overproduction of IL-1*β*, targets for drugs used in the treatment are different. IL-1*β* blocking strategies are the general approach in CAPS, while* colchicine* is the gold standard to reduce the number of FMF flares. Ineffectiveness of colchicine in CAPS suggests that colchicine may have a prophylactic effect only in a specific PC/C range. Low levels of PC and higher levels of activated form of C may correspond to the ineffective range of colchicine, due to inability to use it in higher doses because of potentially fatal adverse effects. Although the mechanism of colchicine is not known yet, colchicine is speculated to suppress Cryopyrin inflammasome activation [46].

**Figure 16:**
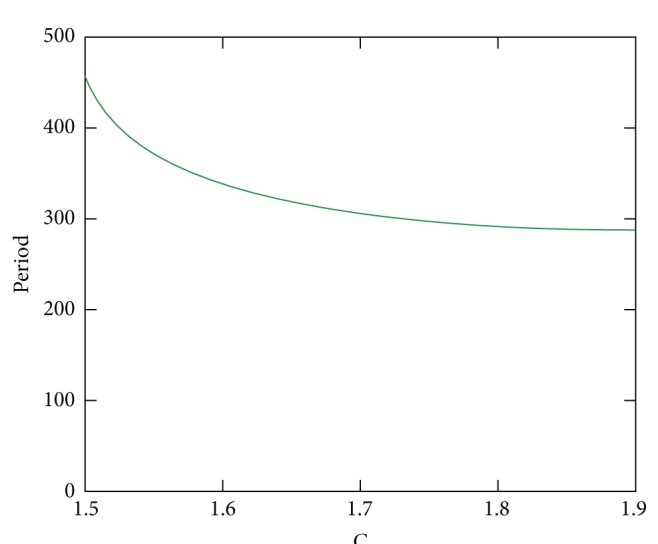
Period of the attacks in CAPS as a function of Caspase 1 level.

Both time-domain simulations and bifurcation analyses justify the classification of model/disease modes we have identified. In Healthy mode, C levels are low, independent of T. In FMF, when T is low, so is C. Higher T levels cause an increase in C values into the excitable mode. In CAPS, C levels are always high, where other key players are in the oscillatory region. Disease severity in the CAPS mode can be correlated with the level of C. Furthermore, Figure 16 shows that the recurrence frequency of the attacks in CAPS increases with increasing values of C.

**Figure 17:**
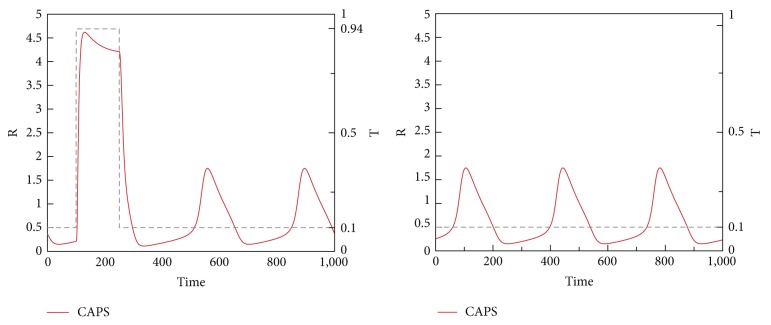
R levels in CAPS with and without trigger.

We have so far observed that CAPS attacks occur even in the absence of a trigger pulse. When there is also a trigger pulse, the attacks become more severe and frequent. Furthermore, C levels may go beyond the end of the oscillatory region, where there is again a monotonically increasing stable solution as shown in Figure 10. Inflammatory response in the CAPS mode with trigger initiation is more severe and this may correspond to the more severe forms of CAPS such as NOMID, in which continuous inflammatory activity is rule. R levels for the CAPS mode in response to a trigger pulse are shown in Figure 17. Base trigger level case is provided for comparison.

## 8. Conclusion

We have presented a unified mathematical model for two of the well characterized autoinflammatory syndromes, associated with increased innate immunity related inflammation, FMF and CAPS. These diseases are caused by the mutations in the regulatory inflammasome proteins, Pyrin and Cryopyrin, respectively. Although the mutations and their inheritance patterns are different, FMF and CAPS are closely related in terms of their pathogenic pathways. Overproduction of IL-1*β* due to increase in Caspase 1 activity is the main cause that triggers the inflammation process in both diseases. That is why we have endeavoured to develop a unified model in order to capture both FMF and CAPS, in addition to the healthy immune system behavior, by adjusting only three parameters in the model. By introducing the effect of relevant mutations, we were able to mimic, in the model, the observed clinical behaviors in terms of recurrence rate, triggers of inflammation, and disease severity which may elucidate the differences in the disease mechanisms. According to the bifurcation analyses performed and simulation results obtained from the model, Caspase 1 level is the most critical parameter in determining the three modes that the model exhibits, Healthy, FMF, and CAPS. In accordance with the clinical literature, FMF comes out as trigger-dependent while CAPS is mostly due to autonomous self-dynamics of the immunity related protein concentrations. As a result, FMF attacks occur only when a trigger or insult is introduced to the system. CAPS, on the other hand, has an autonomous periodic nature, and even when there is no trigger present, attacks occur, if not treated. The model proposed in the paper matches and explains such clinical observations, as distilled from the results presented in the paper and summarized below.

*(i) Clinical Observation 1.* FMF attacks occur irregularly, possibly as a result of external or endogenous triggers such as stress, heavy exercise, and infections.

*Corresponding Model Behavior and Outcome.* FMF attacks are stimulated by a pulse trigger (T) in the model. In Pyrin mutants (i.e., FMF patients), the introduction of a trigger with a large enough magnitude and duration results in higher Caspase 1 levels and a transition from monostable behavior to excitability. This causes increases in the amount of the key variables (i.e., free (I) and bound IL-1*β* (R) and the antagonist (A)), indicating an attack period. When the trigger is removed from the system, the amounts of these key players settle back to their normal values, indicating a remission period.

*(ii) Clinical Observation 2.* FMF attacks have irregular periodicity, ranging from weekly attacks to ones that occur once in several months.

*Corresponding Model Behavior and Outcome.* In the FMF mode of the model, the attack characteristics are determined by the magnitude and the duration of the introduced trigger. Attacks in this mode usually do not have a regular pattern.

*(iii) Clinical Observation 3.* Disease and attack severity exhibits considerable variability among FMF patients. Furthermore, the different attacks experienced by a particular patient may be of varying severity.

*Corresponding Model Behavior and Outcome*. Person to person variations in the attack durations and severity and diverse attack patterns in FMF patients can be explained by irregular trigger actions based on environmental or seasonal variations. This can also be explained based on the difference in the sensitivity to external triggers among individuals along with the penetrance of the mutation, which can be reflected in the model by adjusting the values of the three key model parameters.

*(iv) Clinical Observation 4.* CAPS arise as recurrent episodes of fever, even in the absence of any insult; that is, CAPS flares seem to be self-sustaining. CAPS is a more severe disease than FMF with more frequent and relatively regular attacks.

*Corresponding Model Behavior and Outcome.* When the parameter (that corresponds to the mutation in Cryopyrin) is increased, oscillatory behavior in the key players of the immune system is observed even without any trigger. In other words, CAPS attacks become inevitable.

*(v) Clinical Observation 5.* CAPS is a spectrum of diseases with varying severities. In more severe forms of CAPS, such as NOMID, there is continuous inflammatory activity.

*Corresponding Model Behavior and Outcome.* Our model captures the disease severity in CAPS in two ways. First, even when there is no trigger, the system variables are in oscillatory region due to constant high Caspase 1 levels. The period of the attacks decreases when the degree of penetrance of the mutations in Cryopyrin is increased, as shown in Figure 16. When also a trigger is introduced into the system, the model exhibits a constant, that is, nonoscillatory, but much stronger response, which corresponds to a continuous inflammation and fever as observed in NOMID.

*(vi) Clinical Observation 6*. Although FMF and CAPS are similar diseases in terms of inflammasome-associated overproduction of IL-1*β*, targets for drugs used in the treatment are different. IL-1*β* blocking strategies are the general approach in CAPS, while* colchicine* is the gold standard in FMF in reducing the flare frequency and the symptoms.

*Corresponding Model Behavior and Outcome.* Due to the mutation in Pyrin in FMF, Pyrin cannot suppress inflammasome formation by binding to procaspase 1 and Caspase 1. In FMF, procaspase 1 level and procaspase 1/Caspase 1 ratio are higher due to the effects of mutations in Pyrin on inflammasome regulation. Because of trigger-dependent irregular attack development in FMF, procaspase 1 is the dominant form found in the cytoplasm during attack-free periods, and it is overprocessed into Caspase 1 with trigger, which exceeds physiologic control mechanisms and results in inflammatory attacks. Colchicine is effective as a prophylactic treatment by possibly showing its efficacy when procaspase 1/Caspase 1 ratio is high, and it has no effect during an attack. In CAPS, as a result of the dominantly inherited gain-of-function Cryopyrin mutations, which result in spontaneous activation of the inflammasome, conversion of procaspase 1 to Caspase 1 is highly favored. Therefore, procaspase 1 level and procaspase 1/Caspase 1 ratio are lower in the CAPS mode. Colchicine has no efficacy in CAPS patients, and this clinical observation may also suggest that colchicine's prophylactic efficacy can be observed only in a limited procaspase 1/Caspase 1 range. Low levels of procaspase 1 and higher levels of activated Caspase 1 may correspond to the ineffective range of colchicine, due to its inability to control higher Caspase 1 activity and its limited use at higher doses because of potentially fatal adverse effects. Although the mechanism of colchicine is not known yet, colchicine is speculated to suppress overall Cryopyrin associated inflammasome activation [46] and hence suppress conversion of procaspase 1 into active Caspase 1. This is effective in FMF due to higher procaspase 1 levels, but not in CAPS, because procaspase 1 is already depleted in CAPS according to the model (i.e., converted into Caspase 1). A possible inflammasome-related mechanism for colchicine as a procaspase 1 inhibitor also emerges from the model.

The proposed model explains the clinical observations, as described above, very well in a qualitative sense, and the model variables do have a direct correspondence to key molecular players in the immune system. Elevations in the amounts of the selected key variables (i.e., free (I) and bound IL-1*β* (R) and the antagonist (A)) in the attack period of both FMF and CAPS are captured by our model. The model also shows the recurrence patterns observed clinically in both diseases: irregular attacks in FMF following the stimuli and more frequent and relatively regular attacks in CAPS even without any stimuli depending on mutation-dependent disease severity. On the other hand, the specific values of the parameters used in our model are not yet based on experimental findings. As such, it is not yet possible to correlate the detailed quantitative outcomes of the model with actual quantitative clinical measurements. At this stage, however, the elevations in the concentrations of the aforementioned key variables act as the initiators of the subsequent cascades, which then result in the elevation of easily measurable inflammation parameters such as CRP and ESR in the attacks. Therefore, the values of the key variables that exceed chosen thresholds are considered as the markers of the attack period. Although the parameters are not physiologically meaningful when considered alone, following the adjustment of the parameters, relative changes in the key variables follow a clinically similar scenario.

In our future work, we will strive to link all model variables and parameter values to* in vivo* clinical observations, measurements from* in vitro* experiments based on cell lines, and also* in silico* experiments. In these* in silico* studies based on data from clinical and* in vitro* studies, we will formulate and run detailed molecular dynamics simulations in order to quantify the interactions of various molecular species involved.

## Figures and Tables

**Table 1:** CAPS types and their symptoms.

Disease	Major symptoms	Severity
FCAS	Cold-induced urticarial rash and arthralgia Fever after exposure to cold	The mildest

MWS	Shared symptoms with FCAS and NOMID Inflammation is seen without provocation Cold, stress, and exercise also trigger the inflammation	Intermediate

NOMID	Neonatal-onset high fever, persistent rash, aseptic meningitis, mental retardation, sensory deafness, papilledema, arthritis with bone overgrowth, and secondary amyloidosis	The most severe

**Table 2:** The parameters used in the model.

	Explanation
*V*_*r*_	Maximum rate of I and A binding to the receptor
*V*_*i*_	Strength of positive feedback
*V*_*a*_	Strength of negative feedback
*V*_*c*_	Maximum rate to form Cryopyrin inflammasome
*V*_*pc*_	Maximum rate of PC production

*K*_*ir*_	Threshold for I to induce R
*K*_*ar*_	Threshold for A to suppress R
*K*_*ri*_	Threshold for R to induce I
*K*_*ra*_	Threshold for R to induce A
*K*_*pcc*_	Threshold for PC to induce C
*K*_*tc*_	Threshold for T to induce C
*K*_*pc*_	Threshold for P to suppress C
*K*_*ppc*_	Threshold for P to suppress PC

*k*_*dr*_	Degradation rate of R
*k*_*di*_	Degradation rate of I
*k*_*da*_	Degradation rate of A
*k*_*dc*_	Degradation rate of C
*k*_*dpc*_	Degradation rate of PC

*k*_*br*_	Basal synthesis rate of R
*k*_*ba*_	Basal synthesis rate of A
*k*_*bc*_	Basal synthesis rate of C
*k*_*bpc*_	Basal synthesis rate of PC

*k*_*c*_	Conversion rate of PC into C
*n*	Hill function cooperativity exponent
*α*	Proportionality constant of C and I

**Table 3:** The parameter values used in the model simulations.

Parameter	Healthy	FMF	CAPS
*V*_*r*_	1		
*V*_*i*_	1.4		
*V*_*a*_	0.17		
*V*_*c*_	1.15		450
*V*_*pc*_	1		

*K*_*ir*_	1		
*K*_*ar*_	1		
*K*_*ri*_	1		
*K*_*ra*_	1		
*K*_*pcc*_	1		
*K*_*tc*_	1		
*K*_*pc*_	1	10	
*K*_*ppc*_	1	10	

*k*_*dr*_	0.2		
*k*_*di*_	0.2		
*k*_*da*_	0.02		
*k*_*dc*_	10		
*k*_*dpc*_	0.5		

*k*_*br*_	0.01		
*k*_*ba*_	0.1		
*k*_*bc*_	1		
*k*_*bpc*_	1		

*k*_*c*_	0.3		
*n*	2		
*α*	0.1		
P	2		
